# Potential Role of Phase Separation of Repetitive DNA in Chromosomal Organization

**DOI:** 10.3390/genes8100279

**Published:** 2017-10-18

**Authors:** Shao-Jun Tang

**Affiliations:** Department of Neuroscience and Cell Biology, University of Texas Medical Branch, Galveston, TX 77555, USA; shtang@utmb.edu; Tel.: +1-409-772-1190

**Keywords:** repetitive DNA, ‘junk’ DNA, repeat assembly, chromosome, chromatin, phase separation, CORE theory, membrane-less organelle

## Abstract

The basic principles of chromosomal organization in eukaryotic cells remain elusive. Current mainstream research efforts largely concentrate on searching for critical packaging proteins involved in organizing chromosomes. I have taken a different perspective, by considering the role of genomic information in chromatins. In particular, I put forward the concept that repetitive DNA elements are key chromosomal packaging modules, and their intrinsic property of homology-based interaction can drive chromatin folding. Many repetitive DNA families have high copy numbers and clustered distribution patterns in the linear genomes. These features may facilitate the interactions among members in the same repeat families. In this paper, the potential liquid–liquid phase transition of repetitive DNAs that is induced by their extensive interaction in chromosomes will be considered. I propose that the interaction among repetitive DNAs may lead to phase separation of interacting repetitive DNAs from bulk chromatins. Phase separation of repetitive DNA may provide a physical mechanism that drives rapid massive changes of chromosomal conformation.

## 1. Introduction

Phase separation is a common physical mechanism by which material organization and morphologies are controlled [1,2]. The transition from one phase to another phase of a material or a mixture of materials results from changes in their organization and is often accompanied by drastic alterations in their morphologies. Phase transition is induced by the changes of specific physical and/or chemical parameters that lead to re-organization of the materials or specific components of the system. These include external parameters such as temperature that controls solid–liquid and liquid–gas transitions, and order parameters that characterize different phases, such as density of molecules in the transitions [1,2]. Phase transition provides a mechanism for coordinated or synchronized alteration of the organization of the population of the materials in the system. 

Recent studies indicate that phase separation plays a key role in intracellular compartmentalization of specific proteins and RNAs to form various structural and functional units in the cytoplasm and nucleus [3]. In contrast to classical cell organelles compartmentalized by membranes (e.g., mitochondria and endosomes), the intracellular compartments formed by phase separation are without membrane barriers and are known under various names such as liquid droplets, membrane-less organelles or assemblages. The membrane-less nature of these organelles allows them to freely exchange their components with the surrounding milieu and assemble/disassemble dynamically according to the change of the intracellular environment. Many membrane-less organelles have been reported, including nucleolus, paraspeckles, nuclear speckles, Cajal bodies and promyelocytic leukemia (PML) bodies in the nucleus, and P-bodies, stress granules, germ granules and mRNA-protein (mRNP) granules in the cytoplasm [3,4]. 

Emerging evidence suggests that inter-molecular interactions among proteins and RNAs are crucial for phase separation-based formation of membrane-less organelles [3,4]. The seeding proteins usually have the potential for multivalency in their interactions with other macromolecules via specific interacting modules and/or weak interactions by disorganized regions in proteins. The initial interaction of the multivalent protein nucleates the phase transition and recruits more macromolecules to form the gel-like network of liquid droplets [3,4]. Apparently, the assembling and disassembling of the liquid droplets are modulated by the concentration of the participating seeding proteins and other macromolecules, the modification of these molecules, ionic strength and other intracellular biophysical conditions that change the properties of the involved macromolecules. 

A significant fraction of human and other eukaryotic genomes is made up of repetitive DNA [5,6]. Based on the fact of evolutional dynamic changes of repetitive sequences, repetitive DNAs are considered as major drivers for structural and functional evolution of the genomes and chromosomes. Repeat elements may evolve to become genes and/or transcriptional regulatory elements such as enhancers and promoters [7]. In addition, some landmark structures of chromosomes such as telomere, centromere and pericentromere regions are concentrated with various repetitive DNAs, indicating a key role of these sequences in formatting chromosomes. Lines of evidence suggest that DNA and chromatins with homologous sequences such as repetitive DNA elements have an intrinsic property/tendency for self-interaction [8,9,10,11,12]. I have formulated the CORE (chromosome organization by repetitive elements) theory to describe the critical roles of repetitive DNA in organizing chromatin folding in the higher-order structure of chromosomes. This theory is based on the singular assumption that repeat interaction/pairing (RP) causes the formation of repeat assemblies (RAs) and an RA-based chromosomal skeleton [8,13,14]. According to this theory, the linear distribution of repetitive DNA elements along the chromatins provides structural codes for chromosomal organization. The concept of repeat-directed chromosomal organization is consistent with multiple lines of evidence [8,13,14]. However, the mechanism by which the activity of numerous repeats in the same family is coordinated is unclear. Given the rapid temporal progress of chromosomal dynamics during cell cycles, a mechanism to synchronize the action of RA formation is desirable so that the RAs among repeat members can be formed in a short time period to support rapid chromosomal conformation changes. In this paper, I propose phase separation of repetitive DNA as a potential mechanism.

The high copy numbers of specific repeats along chromatins may generate a local concentration that is sufficient to cause extensive interactions and phase transition of repetitive DNAs. The possibility of phase separation of repetitive DNA, and the structural consequences of the phase separation on chromosomal organization and conformation dynamics will be discussed. I suggest that the population behavior of repetitive DNA in chromosomes follows the physical law of phase separation. This insight may help explain how repetitive DNA elements can reversibly change the morphology of chromosomes in a short time scale. 

## 2. A Model of Repetitive DNA Phase Separation-Mediated Chromosome Organization

**Figure 1:**
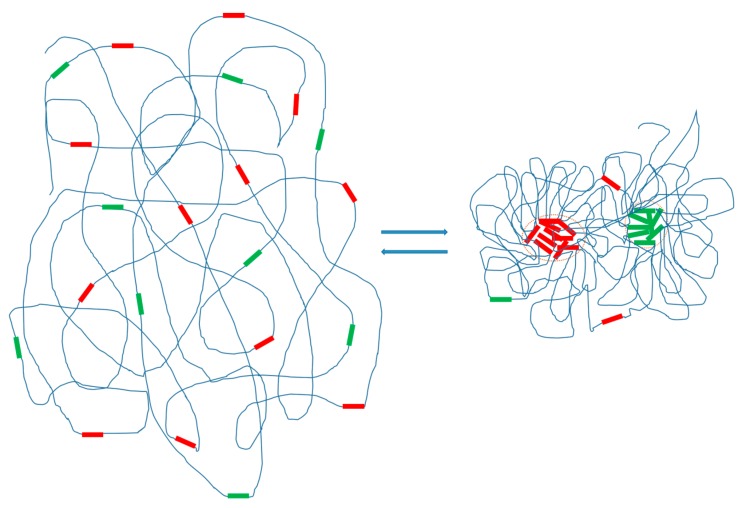
Hypothetical phase separation of DNA repeats in chromosomes. Repeats in two different families (indicated with red and green colors) are used to illustrate the idea of phase transition of repeat DNA in a chromatin segment. Before phase transition (left), the repeats are randomly distributed in the territory of an uncondensed chromosome. After transition (right), repeats are phase-separated from the bulk chromatin and form repeat assemblages (indicated by circles), which likely contain other molecules such as proteins and RNAs. The formation of the assemblages causes chromosome condensation. Assemblages of different repeats may have the potential to merge into bigger chromosomal domains under specific conditions (not shown).

Emerging evidence supports the general notion that seeding proteins with the potential of multivalent interaction with other macromolecules can initiate phase separation and the formation of droplets when a threshold concentration is reached under specific conditions [3,4]. I have previously suggested the potential that repetitive DNAs interact with multiple other homologous sequences (or chromatins) during RA assembly [8,13,14], based on published data, including the observation that many repetitive DNA families have large copy numbers. These properties of repetitive DNAs fulfill the basic requirement for intracellular phase separation. Based on these considerations and the emerging evidence, I propose that specific families of repetitive DNAs will undergo RA formation via phase separation at specific cell states. Phase separation of repetitive DNAs may provide an environment that promotes rapid repeat compartmentalization and RA formation and thus a mechanism for a synchronized completion of RA formation on a large scale (Figure 1). The conceived phase separation of repetitive DNAs might significantly contribute to the process of chromosome organization. I intend to suggest a model that entails the following critical ideas:

(1) Families of repetitive DNAs in chromosomes undergo phase separation. I propose that in the higher-order chromosomal organization specific families of repetitive DNAs tend to phase-separate from bulk chromatin DNA. Both tandem and dispersed families of repeats are expected to undergo phase separation under suitable conditions. However, different types (tandem vs. dispersed) or families (e.g., different families of tandem or dispersed repeats) of repeats may behave differently (see below). Furthermore, I suggest that these phase-separated repetitive DNA elements form intrachromosomal membrane-less organelles—RAs—that are composed of repetitive DNAs and other associated macromolecules such as proteins and RNAs. I have previously summarized the evidence of repetitive DNA spatial clustering in the nucleus and chromosomes, including dispersed repeats and tandem repeats [8,13,14]. In supporting the idea of phase separation of repetitive DNA in chromosomes, recent studies have shown that tandem repeat ribosomal DNAs (rDNAs) form nucleoli by phase separations [15,16]. Another study found that heterochromatins, which are enriched with repetitive DNAs, are also condensed—a mechanism involving phase separation [17].
(2) Repetitive DNA phase separation is initiated by molecular processes that involve RPs and probably other types of intermolecular interactions among proteins and RNAs. I propose that that RP is a critical form of interaction among repetitive DNAs undergoing phase separation. Homology-sensing-based DNA interaction is a general phenomenon, as suggested by evidence discussed previously [8]. Multiple mechanisms of RPs have been suggested, including direct interaction between chromatins with repetitive DNAs and indirect interaction mediated/stabilized by other molecules such as proteins and RNAs [8]. One repetitive DNA may have the potential for multivalent interactions with multiple other homologous repeats via different parts/motifs, different surfaces, and/or different mediators [8].
(3) Repetitive DNA phase separation facilitates the organization of chromosome structures. I conceive that the proposed repetitive DNA phase separation would significantly impact chromosome organization in several ways. First, phase separation of specific families of repetitive DNA elements would create a physical driving force to change chromosomal conformation. Second, phase separation probably creates an environment to synchronize the behavior of the numerous members of repetitive DNA elements in the chromosomes and thus can coordinate the activity of repeats in chromosomal organization on a short time scale. Third, phase-separated RAs may form relatively stable chromosomal structures to support a specific conformation of a chromosome. Finally, the dynamic nature of phase-separated structures such as RAs provides a plastic mechanism for the changes of chromosomal organization during cell cycles and transitions to different cell states with different functions.

## 3. Regulation of the Phase Separation of Repetitive DNA Elements in Chromosomes

Based on the findings of intracellular phase separation in the formation of various membrane-less organelles, I expect that the phase separation of repetitive DNAs is regulated by multiple factors. They include intrinsic and external factors, corresponding to the order and external parameters respectively. 

Critical intrinsic factors include copy number and distribution patterns of repeats. Copy numbers of repetitive DNA elements in a specific family would presumably affect phase separation of that repeat family. Different families of repetitive DNAs vary in their copy numbers and thus can be classified into high-, medium- and low-copy repeats in the genomes [5,6]. The copy number would directly relate to the concentration or density of the element in the nuclear space, and consequently to the interaction of repeats. 

Repeats in a specific family often unevenly distribute in the linear genome [5,6]. For example, tandem repeats often form head-to-tail arrays in specific loci such as pericentromeric regions. Even interdispersed repeats also tend to unevenly cluster in specific chromosomal regions [18]. The distribution patterns on chromatin (such as clustering) of repeats in the same family would obviously affect the local concentration of the repeats in a specific nuclear domain occupied by the chromatin regions. For instance, tandem repeats and clustered dispersed repeated would increase the local concentration of given families and thus promote RA formation and phase separation of the repeats.

External factors, including repeat-interacting molecules, intracellular ions and modification or transcription of repeats, may play significant roles in regulation of phase transition of repeats, by modulating RP kinetics. The sequences of repeat elements likely contain information that directs their interaction with other repeats and molecules such as proteins and RNAs. Different DNA sequences may have different intrinsic tendencies for self-interaction. For example, different sequences may provide unique surfaces for different interaction affinity. The same repeat, especially a long repeat, may interact with different repeats via different segments. In addition, repeats may also contain different binding sites for other interacting ligands (e.g., proteins and RNAs). In these potential ways, repeats may acquire multivalency of interactions with homologous repeats and other molecules to form an interacting network, which is a hallmark for the formation of membrane-less organelles via phase separation. 

DNA repeats are often epigenetically modified [5,6], including by cytosine and histone methylation. Such chromatin modifications on repeats play a key role in suppressing their expression. They likely also change the conformation and biophysical properties of the local chromatin regions and thus affect their interaction capacity with other repeats, proteins and/or RNAs that modulate phase separation.

The intracellular ionic environment is very dynamic and changes according to different cell states. As discussed previously, the ionic strength of various cations such as Ca^2+^ and Mg^2+^ may be crucial in regulating RA-based chromatin folding [19]. I expect that the cell state-based changes of ionic strength would play an important role in regulating the separation of repeats. 

In addition to alterations of ionic strength, different cell states may change other critical factors that modulate repeat phase separation. For instance, different cell states may synthesize (or change the expression levels of) different proteins that can bind to repetitive DNA elements to modulate RA formation.

It is well known that many repeats can be transcribed, especially in response to various stresses. These transcribed repeats include interdispersed and tandem repeats [20,21,22,23]. Transcription has been suggested as a critical mechanism for the formation of certain chromosomal membrane-less organelles such as nucleoli [16]. I conceive that the transcription states of repeats will have a significant effect on repeat phase separation, and co-activation of transcription of repeats in the same family would promote their agglomeration.

## 4. Novel Features of the Model

There are several novel features in the current model. First, the model suggests the possibility of intramolecular phase separation. Previous studies of phase separation-mediated intracellular organization have focused on membrane-less organelles that are formed by inter-molecular interactions, seeded by proteins with the capacity for multivalent interactions. Here, I propose that phase separation of repeat DNAs occurs intra-molecularly (intra-chromatin) during chromosomal organization. 

Second, the current model suggests that intra-chromatin phase separation of repeats plays an important role in organizing chromosomal conformation, and phase-in and phase-out of repeats may drive the dynamic alterations of chromosome conformation. Previous models of intracellular membrane-less organelles mainly describe the organization of specific cellular components such as proteins, based on inter-molecular interactions. In contrast, the current model describes the role of repeat phase separation in organizing the conformation of a single chromatin fiber. 

Third, the phase separation described in the current model may provide a potential mechanism for synchronizing repeat action in RA assembly during chromosome organization. The current model does not change the basic ideas of the CORE theory [8]. According to the CORE theory, numerous RPs (and thus RAs) are expected to contribute to the organization of a specific conformation of a chromosome. Because a switch of chromosome conformation can be completed a relatively short period of time in vitro and during cell cycles, a mechanism needs to be in place to promote the completion of RA assembly and/or disassembly in a synchronized manner. The idea of phase separation of repetitive DNA elements proposed here may provide such a mechanism.

## 5. Considerations of Potential Problems

Although I propose here that repeats have an intrinsic tendency for phase separation and that the phase separation of repeats has a role in organizing chromosome structures, some key questions remain to be answered. For example, in genomes of higher eukaryotic organisms (e.g., mammals), there are many different repeat families. It is not clear how the behavior of phase separation would differ between various types of repeat families or whether repeats in all families have the capacity for phase separation. Nonetheless, because repeats in different families are intrinsically different in aspects that may critically relate to phase separation (as discussed above), including their sequences, distribution patterns on chromatins and copy numbers; I expect that they likely show different behaviors in their phase separation. For instance, repeats in different families may undergo phase separation in different stages of the cell cycle. Different physiological states of the cells that associate with different sets of intracellular conditions that modulate repeat phase separation—such as cation concentration and the expression levels of repeat interaction proteins and/or RNAs—may also play a role. The conceived differences in phase separation characteristics of repeats in different families are likely coupled with different functional states of the cells or are in response to different external stimuli such as stress or other environmental factors. Under a specific condition or cell state, repeats in some families are phase-separated, while some others may be mixing with bulk chromatins. The conformation of the chromosome under different cell states is governed by the repeats that are phase separated at those states. The transition of chromosomal conformation in different cell states may be associated with both the phase separation of some repeat families and the loss of phase separation of other families, which may allow them to mix with bulk chromatins. The combination of the two processes of different repeat families could drive the switch of chromosomal conformation.

I think that RPs are critical for the phase separation of repeats. However, I do not exclude other forms of molecular interactions that may be involved in the phase separation of repeats. The involvement of molecules such as proteins, RNA and transcription factors that facilitate or modulate RPs is highly probable. As suggested in the bacterial ParABS system, protein-DNA complex formation may facilitate phase separation of DNA [24]. The future characterization of the molecular composition of RAs will help further our understanding of repeat phase separation-mediated changes in chromosomal conformation. Although I conceive that RP-mediated separation is a thermodynamically driven process, it is unclear whether recruiting specific macromolecules such as proteins to the phase-separated repeat assemblages involves active processes, as recently identified in other systems [25,26]. 

In the CORE theory [8], I suggest that RP-based RA formation is a molecular mechanism that underlies the organization of chromosome conformation. Here, I propose a role of repeat phase separation in chromosomal organization. It is important to point out that these suggestions do not contradict each other in nature. In fact, I think RA formation is a process that underlies the phase separation of repeats. On the other hand, phase separation of repeats probably generates an ideal environment for the formation of RAs; while the loss of phase separation may disrupt the environment that supports the formation and stabilization of RAs and cause rapid massive disruption of RAs. 

I conceive repeat phase separation as a major potential physical mechanism that drives the organization or plasticity of specific chromosomal conformations. This suggestion is based on multiple considerations. First of all, phase separation may facilitate numerous weak interactions among homologous repeats, and the massive action of repeats is expected to drive instantaneous conformational changes. Second, analogous to the role of weak interactions such as hydrophobic interactions in protein folding, weak interactions among homologous repeats facilitated by phase separation may also play a crucial role in chromatin folding in chromosome organization. Third, the phase separation of repeats would cause their de-mixing from the other chromatin regions in the same chromatin fibers, and this might naturally bury the phase-separated repeats and their complexes (RAs) in bulk chromatins to form skeleton-like structures in chromosomes. 

Emerging evidence from studies on phase separation during the formation of nucleoli and heterochromatins supports the idea of repeat phase separation in chromosomes [16,17]. To directly test this hypothesis, further experimentation is needed. For example, it would be interesting to demonstrate phase separation of repeats using in vitro assays and determine the conditions for the phase separation of different repeat families. Furthermore, it would be critical to show the phase separation behavior of specific repeat families in cells. 

## 6. Conclusions

Based on the intrinsic property of repetitive DNA in homology-sensing-based interactions, I propose that they are subjected to phase separation in the nucleus under suitable conditions. Although this idea remains to be tested directly, it is consistent with the emerging evidence from the observations of phase separation of rDNA and heterochromatins that are associated with or contain high amounts of repetitive DNAs [16,17]. In addition, repetitive DNAs are often spatially clustered [8,27]. Phase separation has been considered as a primary mechanism for compartmentalizing the intracellular space to form functional assemblages (i.e., membrane-less organelles). Our model expands this conceptual framework by extending the role of liquid–liquid phase separation in chromosomal organization. In contrast to many membrane-less organelles that are formed from phase separation, this is intra-chromatin phase separation in nature. Phase separation provides an attractive physical mechanism to coordinate the partitioning and condensing of repetitive DNAs (repeat assemblies/RAs) to form the proposed chromosome skeleton that supports the structure of chromosomes. Evolutionary changes of copy numbers and distribution patterns of repetitive DNAs on chromatin may have a significant impact on chromosomal organization by modulating their phase separation behavior.
